# A mechanical model based on watermelons for the study of dynamic cranial remolding orthoses

**DOI:** 10.1038/s41598-025-12088-2

**Published:** 2025-11-28

**Authors:** Fernando Veloso, Pedro Morais, Helena Torres, Anne Fritze, Mario Rüdiger, Gabriele Hahn, A. C. M. Pinho, Jorge Correia-Pinto, João L. Vilaça

**Affiliations:** 1 2Ai – School of Technology, IPCA, Barcelos, Portugal; 2 LASI - Associate Laboratory of Intelligent Systems, Guimarães, Portugal; 3 Department for Neonatology and Pediatric Intensive Care Medicine, Medizinische Fakultät, Children’s Hospital, TU Dresden, Dresden, Germany; 4 Institute for Radiological Diagnostics, Medizinische Fakultät, TU Dresden, Dresden, Germany; 5 Algoritmi Center, School of Engineering, University of Minho, Guimarães, Portugal; 6 Department of Mechanical Engineering, School of Engineering, University of Minho, Guimarães, Portugal; 7 Life and Health Sciences Research Institute (ICVS), School of Medicine, University of Minho, Braga, Portugal; 8 ICVS/3B’s -PT Government Associate Laboratory, Braga/Guimarães, Portugal; 9 Department of Pediatric Surgery, Hospital of Braga, Braga, Portugal

**Keywords:** Deformational Plagiocephaly, Cranial remodeling orthosis, Biological Phantom with watermelons, Biological physics, Paediatric research

## Abstract

Deformational plagiocephaly is a head deformity in newborns that can be treated in some cases with cranial remodeling orthoses that constrain head growth to reshape it. To mitigate complications arising from the treatment with these orthoses, and for the exploration of novel functionally graded lattice structures, a biological model that mimics some aspects of human head growth could provide a development and testing platform for these lattices. In this work, we propose a novel biological model of infant heads in which watermelons are used for the study of cranial remodeling orthoses during head growth and the correction of deformities. First, we reshaped ten watermelons with infant head shapes with deformities via custom molds, which were generated from MRI scans of infants with head deformities. The shaped watermelons were subsequently compared with the original head scans to assess the accuracy of the process via standard clinical measurements. Finally, the growth of four of these watermelon shapes was monitored after the molds were left for several days. The watermelon head shapes registered an average shape difference from the original models of 1.6 millimeters, with a standard deviation of 1.88 millimeters. After leaving the molds, the shapes continued growing, maintaining the ability to be reshaped by external physical constraints. By mimicking two key mechanical aspects of head growth in newborns — growth and deformability — this preliminary approach to a biological phantom offers a promising platform for studying the mechanical behavior of novel lattice structures in the development of cranial remodeling orthoses.

## Introduction

**Fig. 1:**
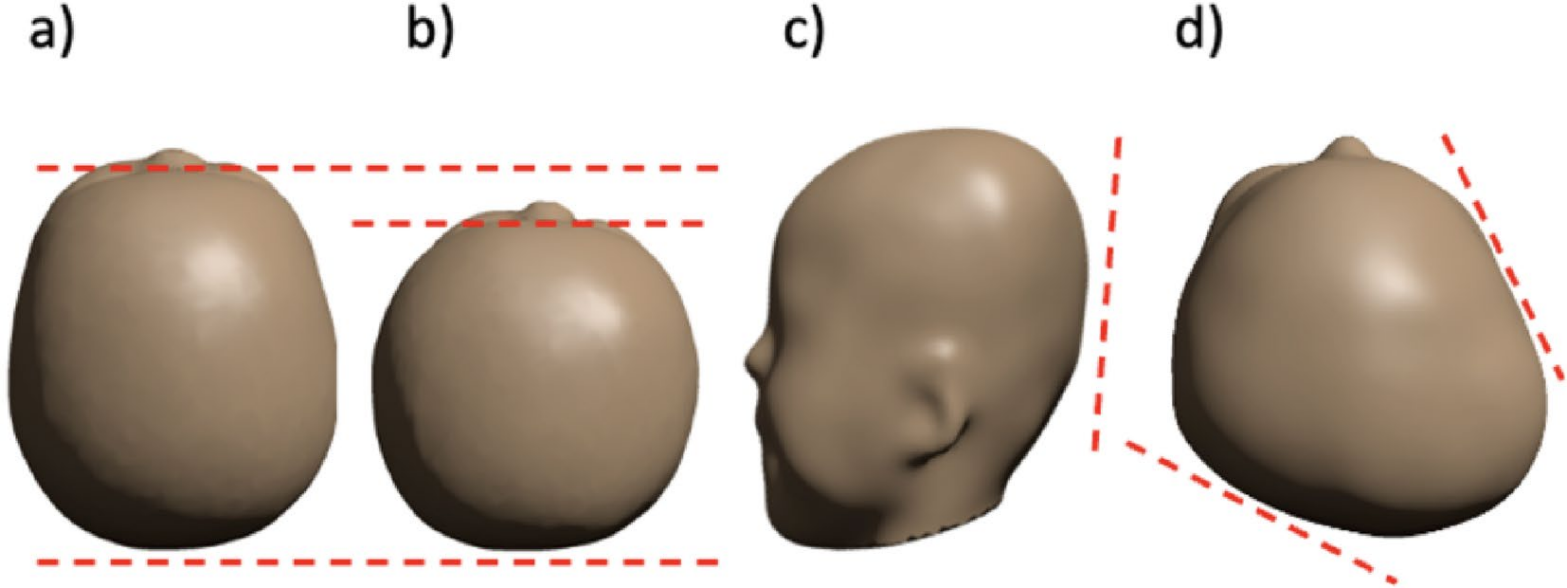
Typical shapes of heads with deformational plagiocephaly. On the left, a comparison of the geometry of a normal head (**a**) with a head shape with deformational plagiocephaly (**b**), brachycephaly (**c**) and **d**), top view of asymmetric deformational plagiocephaly. The 3D models in this figure were generated from MRI images acquired from the Department of Neonatology and Pediatric Intensive Care Medicine, University Hospital Carl Gustav Carus (Medical Faculty of TU Dresden, Germany). The 3D models were created through a custom segmentation method implemented in MATLAB, previously developed by Torres et al. This method, described in detail in a prior publication^11, ^segments head structures from magnetic resonance imaging (MRI) data to produce the 3D representations illustrated in this figure.

 Deformational plagiocephaly (DP) is a head deformity that occurs in newborns and originates mainly in the first months of life. The prevalence of DP can reach 46% at three months and 27% at six months of life^1^. DP is characterized by asymmetry of the head in the sagittal plane and flattening of the occiput, as depicted in Fig. 1. The risk of DP is significantly increased in prematurely born or sick newborns or infants lying predominantly in the same position in the first months of life. This cranial deformation may impede the infant’s natural psychomotor development and may impact cognitive and motor abilities later in life^2^.

There are several forms of treatment for this condition, which are applied according to the degree of severity and age of the patient. When it is found at an early stage and the degree of asymmetry is low, DP can be treated with periodic repositioning of the head in different positions or with physiotherapy. In cases where these treatments are not effective because the degree of asymmetry is high or the infant has an advanced age that will not allow its normal recovery by growth, treatment with a cranial orthosis (CRO) is prescribed^3^.

The rapid growth and flexibility of an infant’s skull increase the risk of deformation^4^. Around seven weeks of age, the prevalence of this condition can reach as high as 46.6%^5^, decreasing to about 3.3% by the age of two^6^. The recommended treatment window typically spans from five to nine months of age, sometimes extending until craniosynostosis (fusion of the skull plates) occurs, after which helmet therapy becomes ineffective. During this period, the skull grows rapidly, and the bone plates remain unfused—ideal conditions for reshaping the head. However, this also presents a challenge for orthotists: current helmets consist of a rigid outer shell and a soft inner foam layer up to twelve millimeters thick. While the rigid structure is necessary to apply consistent pressure, it limits adaptability. As the skull grows, orthotists must frequently scrape away foam to accommodate the changes, but eventually, the helmet becomes too small.

Complications often result from a lack of parental training and insufficient caregiver support in adhering to the wearing schedule and monitoring the orthosis fit. Other reported issues include increased sweating, pain, unpleasant odors, erythema from alcohol-based disinfection, poor orthosis adaptation, skin infections, and reduced physical closeness between parents and child^7,8^.

To explore different strategies for treatments in a safe environment, models that may mimic some aspects of the natural course of head growth in newborns are needed. Some descriptive models based on statistical analysis and comprehensive models using finite element or diffusion models have been described in a recent review by Geoffroy et al.^9^. Weickenmeier et al. developed a numerical prediction of skull shape by modeling physical constraints on skull growth on the basis of different types of synostoses^10^. A statistical shape model was developed by Zhang et al. to predict the shape of infants’ heads via a dataset of 793 children’s head scans^12^. Zhigang et al. used 59 head computed tomography (CT) scans to create a statistical skull geometry model for children up to 3 years of age^13^.

A computational model was developed by Libby et al. to model the growth of the infant´s head during the first year of life^14^. Here, the authors also produced a 3D printed in vitro model with a balloon inside, filled with different amounts of water to create an expansion, simulating head growth. However, this model uses rigid bone plates, and skull growth is replicated mainly by filling the balloon. Thus, the main limitation is the absence of the typical reshaping of the bone plates that occurs during growth. Jing et al. presented a hybrid computational model for simulating craniosynostosis during infant skull development by incorporating finite element and volume-preserving structural modeling to study the interaction between the growing brain and the skull^15^. Barbeito-Andrés et al. modeled the effect of brain growth on the displacement of cranial bones via finite element analysis and geometric morphometrics^16^.

The motivation for the present work comes from a previous publication that explored the dynamic compression behavior of a three-dimensional structure produced via additive manufacturing, designed to influence the growth of infant heads^17^. In the present work, to enable experiments with these lattice structures in future works, we investigate what we identified as a possible mechanical analog—a living, growing watermelon—due to its ability to be reshaped by external forces while undergoing growth.

For this, we consider the use of this biological model. Biological models are important for the validation of medical devices because they can mimic some of the complex phenomena of human tissues and organs, allowing researchers to test the interactions with these biological systems in a safe, controlled environment. With these models, it is possible to assess the performance of devices while minimizing ethical concerns.

**Fig. 2:**
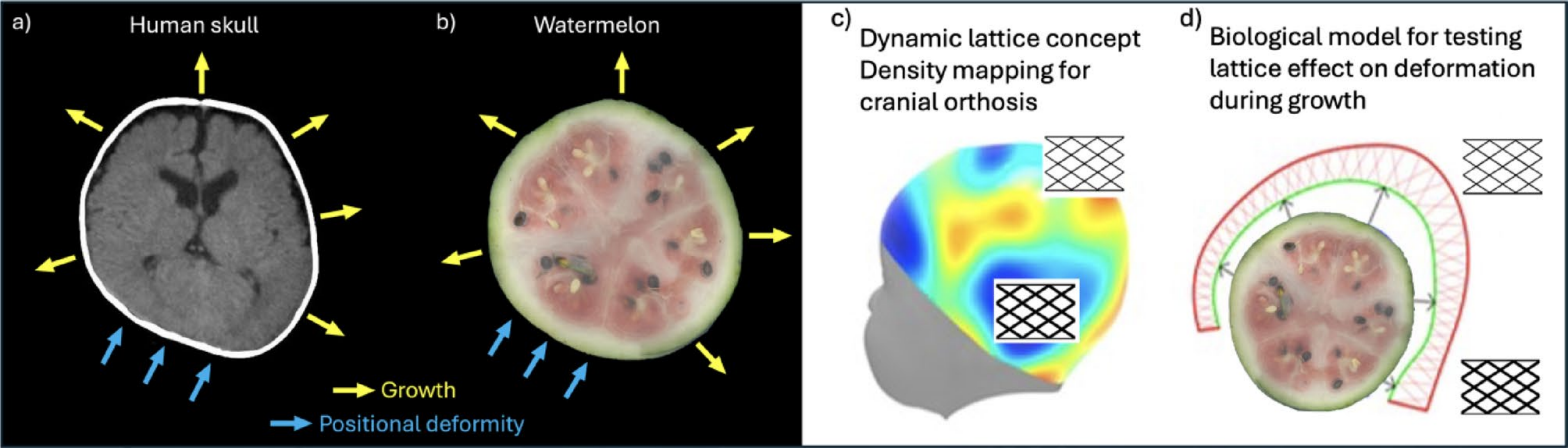
On the left, a section view of an infant´s head and a watermelon. In **a**), the human head grows in all directions and is deformable by external constraints over time, during growth. In **b**), the watermelon also is capable of growth in all directions and is also affected by external constraints that may cause flattening of its surface. On the right, in **b**), the concept for a cranial remolding orthosis, with a functionally graded lattice structure with custom lattice density distribution, to be studied in future works. Finally, in **d**), the aim of this work, to create a watermelon-based head shape, able to grow and be deformed inside the novel lattice structures, serving as a preliminary testing and development platform. Figures **c**) and **d**) are adapted from^16^ with permission from the author.

The biological model presented in this work aims to create an experimental platform to study the performance of a novel cranial remodeling device in more detail, as shown in Figs. 2c) and 2 d). The device is a customized helmet that uses a compressible lattice structure inside, and it is important to understand how it behaves when it is compressed. To make this device more effective and comfortable for patients, we need to study how the lattice structure changes when head growth pressure is applied. To achieve this, in this work, we experiment with the watermelon as a biological growth model with analogous growth and deformability as shown in Figs. 2a) and 2b) that can replicate some of the growth dynamics occurring in the infant’s head from a mechanical perspective.

 The rest of this paper is structured as follows. In Sect. 2, the methods used to select the biological model, obtain the head shapes, and manufacture the molds with these shapes for the biological models are described. In this section, the cultivation and measurement of the watermelons are described. In Sect. 3, the results are presented and discussed in Sect. 4. Finally, further refinements are proposed, and conclusions are drawn in Sect. 5.

## Methods

### The watermelon as an experimental platform for growth and deformability

Watermelons are known to be easily reshaped during their growth by inserting them into molds. In the medical field, they are used as quality assurance phantoms for cranial stereotactic radiosurgery treatments in cancer patients^18^. Watermelons have also been studied for the planning of deep brain stimulation and used as phantoms in preoperative magnetic resonance imaging experiments^19^. However, to the best of our knowledge, mechanical studies with watermelons as biological models for human growth have not been reported in the literature. Thus, in this work, we explore the possibility of using watermelons as simplified biological growth and deformability models, aiming to create a platform in future works for tests with novel dynamic cranial remolding orthoses.

Three main reasons were identified for the selection of watermelon. The first reason is that some watermelons present a flattened region corresponding to the area that is in contact with the ground. These flat surfaces are often yellow due to low exposure to light caused by prolonged periods at the same position during growth, and growers sometimes rotate watermelons to avoid the appearance of asymmetrical shapes. This deformation process could be considered geometrically analogous to the deformity of an infant’s head, in cases where it is caused by prolonged positioning on one side during the first months of life, which is one of the causes of deformational plagiocephaly^3^. Second, the watermelon grows in all directions to an ellipsoidal shape, and it can be constrained during growth by placing it inside a rigid mold to achieve the desired shape. The rigidity of the outer rind (exocarp) allows the use of rigid molds, and three-dimensional (3D) scans can be performed on its smooth surface. The third reason for watermelon selection is size and growth speed. A watermelon can reach the size of a 6-month-old infant’s head in approximately four to six weeks, and reshaping experiments can be performed in a relatively short period, usually within a week.

### Method overview

**Fig. 3:**
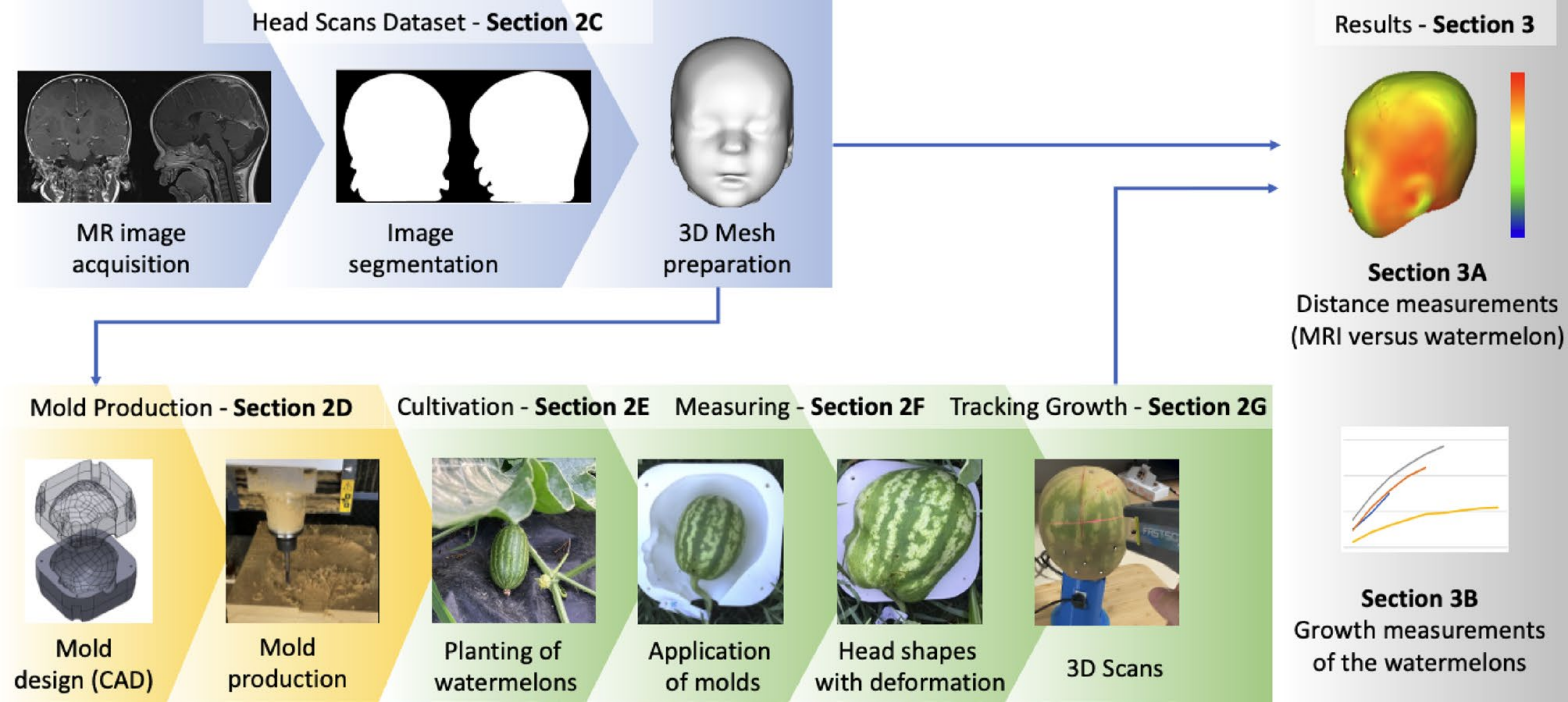
Overview of the method used in this work. MRI images (top left) from the Department of Neonatology and Pediatric Intensive Care Medicine, University Hospital Carl Gustav Carus (Medical Faculty of TU Dresden, Germany). 3D image (top right) generated with Cloud Compare *software* (version 2.12.4) [GPL software] (2021) retrieved from http://www.cloudcompare.org/). 3D model of mold designed in Solidworks version 2020 (*Solidworks*, SolidWorks Corporation, Dassault Systèmes – www.solidworks.com) and photos in bottom row created by the authors.

In Fig. 3, an overview of the method used in this work is presented. In Sect. 2 C, ten head shapes were acquired, and 3D meshes were produced. These meshes were used to design and produce molds for shaping watermelons during growth, as described in Section 2D. In Section 2E, a field was subsequently cultivated with watermelons, and when these achieved a specified size, they were subsequently placed inside the produced molds until they achieved the desired shape. For the measurement and three-dimensional scanning of the watermelons, a method is described in Section 2 F, and the tracking of the growth is described in Section 2G. In Sect. 3, the results are presented. A comparison is made between the original 3D meshes and the produced watermelons by measuring the distances between them and is described in Section 3 A. In Section 3B, after the watermelons were shaped, four of them were selected to perform daily scans while growing freely outside the molds for a few days.

### Head scans dataset

Ten head shapes of newborns were acquired from the Department of Neonatology and Pediatric Intensive Care Medicine, University Hospital Carl Gustav Carus (Medical Faculty of TU Dresden, Germany), with different levels of DP, brachycephaly, or a combination of both.

The ten head shapes originated from magnetic resonance (MR) images taken of infants from February 2003 to August 2018. The data were obtained during routine clinical procedures, and all patients had clinical indications for diagnostic MR. Image acquisition was performed via a 1.5 T MR system (MAGNETOM Symphony 1.5 Tesla MRI System, Siemens, Munich, Germany) with pediatric head coils (*in* vivo). The head images were acquired from the MRI with the isotropic pixel spacing of 0,5 mm, and no contrast enhancement was used. The data were anonymized for further processing. The heads were semiautomatically segmented in the MR images via a simple threshold method and then refined via mathematical morphology. This created 3D shapes for the design of the molds. When necessary, manual adjustments of imperfectly segmented 3D models were carried out.

### Design and manufacture of the molds

From the ten MR images of the infants’ heads, three-dimensional surface meshes were generated and exported in stereolithography (STL) format. The models were processed with the software Meshmixer (*Meshmixer*. Autodesk, Inc.; 2018.) and converted into volume meshes for further processing.

**Fig. 4:**
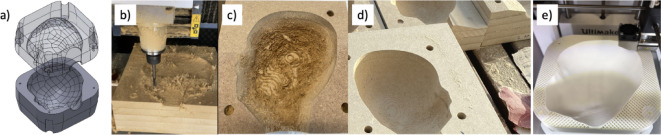
Design and manufacture of the molds for the reshaping of the watermelons. **a**) three-dimensional model of the two mold halves (author´s own work, designed in Solidworks 2020, (*Solidworks*, SolidWorks Corporation, Dassault Systèmes, www.solidworks.com/), **b**) CNC milling of a mold cavity, **c**) milled cavity with residual roughness, **d**) sanded cavity, and **e**) additive manufacturing of a mold cavity.

The head shape molds were designed in the computer-aided design (CAD) software SolidWorks 2020 (*Solidworks*, SolidWorks Corporation, Dassault Systèmes) via 3D volume meshes of the infant’s heads. For the design of each mold, the head shape was subtracted from a cube to produce the corresponding negative shape and divided into two mold cavities by the sagittal plane of the head. An opening for the watermelon stem was added to both sides (Fig. 4a). The two halves are aligned with four wooden dowel pins in the corners and are joined together with F-clamps. Using the designed 3D models, the mold halves were produced via two different manufacturing processes for logistical reasons. Some molds were produced via computer numerical control (CNC) milling of blocks made of 16 mm thick medium density fiberboard plates with a height and width of 230 mm glued together (Figs. 4b), 4c), and the milled surface was finished by manual sanding (Fig. 4d)). The CNC milling machine used was a Roland MDX 540 (Roland Corporation). The remaining molds were produced via additive manufacturing via an Ultimaker 2 Plus 3D printer (Ultimaker B.V.), which uses polylactic acid (PLA) with an infill of 20% and a shell of 2 mm (Fig. 4e).

### Cultivation of watermelons

**Fig. 5:**
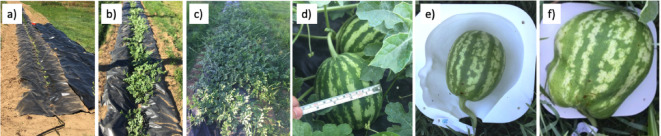
Cultivation timeline. **a**) planting at day 1, **b**) day 25, **c**) day 35, **d**) day 43, **e**) day 43, the watermelon achieved the desired dimensions and is placed inside the mold, and **f**) day 49, the resulting shape of the watermelon.

The cultivation of watermelons started at the beginning of June 2021 in northern Portugal. Initially, the soil was tilled and prepared with nitrogen-based fertilizer. The Crimson Sweet variety was selected for planting at a 0.5 m distance between each plant. The seedlings were acquired from Casa dos Pintos (www.casadospintos.pt). An automatic watering system was used for drip irrigation every twelve hours for periods of fifteen minutes (Figs. 5a) to 5c). The temperature in this region of Portugal during June 2021 remained consistently between 14 °C at night and 23 °C during the day, with no cloudy or cold days. These stable conditions ensured homogeneous watermelon growth rates. A black plastic sheet was used to cover the ground to avoid the growth of weeds and conserve soil moisture.

As the watermelons reached a diameter of approximately 80 mm to 100 mm (Fig. 5d), they were placed inside the ten respective molds (Fig. 5e). The molds are intended to shape the watermelon to the original shape of each infant’s head, replicating the initial deformity. To assess when the watermelons filled the mold cavity, a daily inspection was performed (Fig. 5f).

### Measurement and analysis of the resulting head shapes

**Fig. 6:**
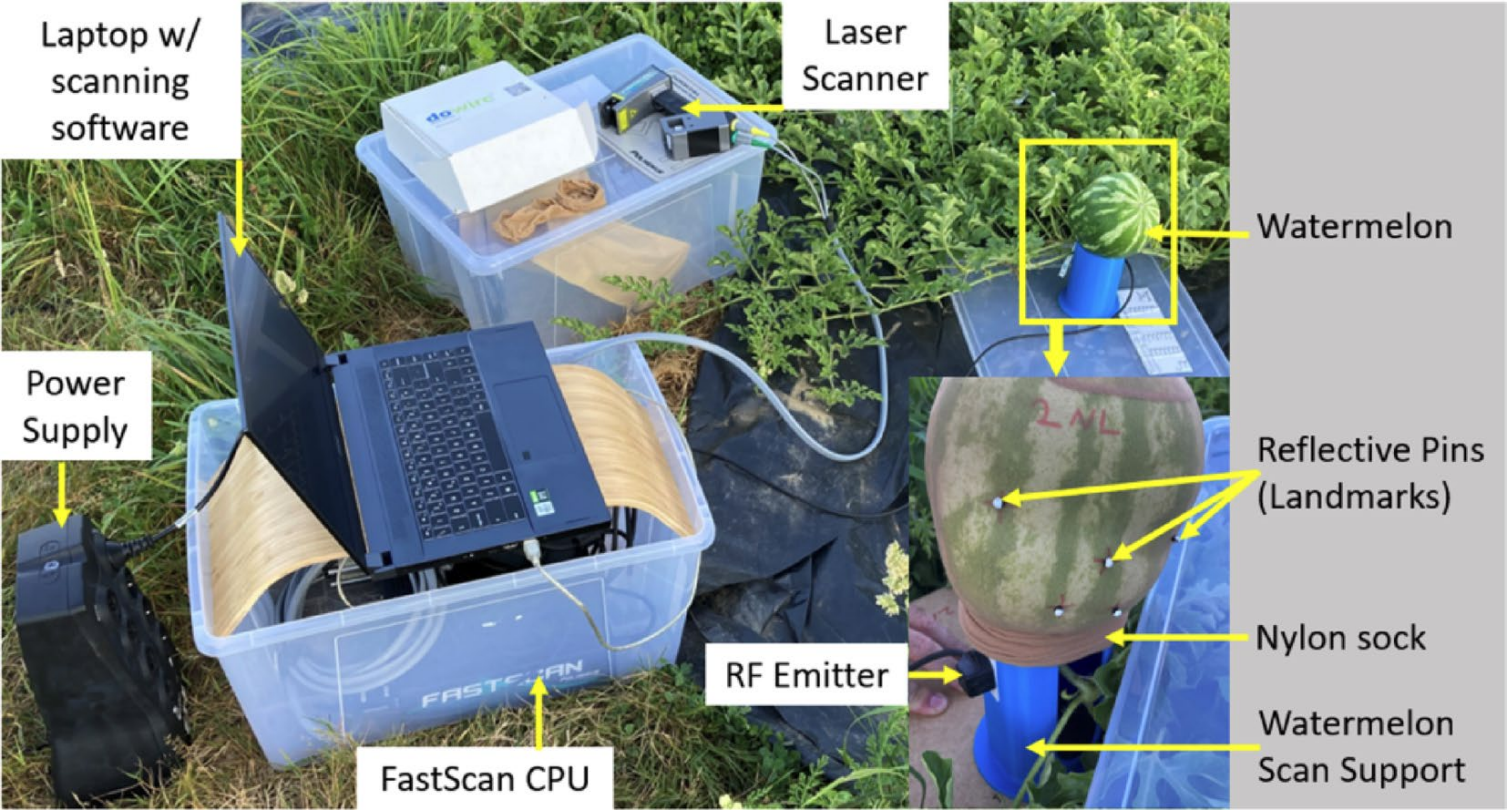
Setup of the 3D scanning of the watermelons.

To verify the similarity between the original 3D scans and the produced head shapes, the watermelons were removed from the molds and scanned with a three-dimensional laser scanning device *(FastScan Cobra*, Polhemus, Inc.). During the scanning process, all the watermelons were covered with a nylon sock for better surface data acquisition and placed on a specially designed support for the fixation of the watermelons and the RF emitter. The setup of the scanning procedure is shown in Fig. 6.

For the analysis of the shape of the ten watermelons, the 3D scans were compared to the 3D model of the original head scan performed on the infants via Cloud Compare *software* (version 2.12.4) [GPL software] (2021) retrieved from http://www.cloudcompare.org/). The 3D scans of the watermelons were aligned with the original 3D models via the five reflective landmarks depicted in Fig. 6, bottom right corner, which were placed on the exocanthions (the outer side of the eyes), the pronasale (nasal tip), and the oral commissures (ends of the mouth).

For the registration of the point cloud pairs, the iterative closest point function (ICP) was used. The resulting point clouds were measured from the nearest points of each mesh, and their Euclidean distances were computed. For each pair of MRI scans and watermelon scans measured 3D point cloud distances, the mean distance and standard deviation were calculated. In the clinical measurement context, our focus on developing customized cranial orthoses (specialized helmets), led us to investigate accuracy standards in the literature. Research indicates that measurements of infant cranial structures can exhibit standard deviations of up to 3 mm when quantifying diagonal difference (DD)^20^.

Additionally, symmetry-related measurements were taken from all the 3D scans that are typically used in the diagnosis of DP: the cranial ratio (CR) and the cranial vault asymmetry index (CVAI) via a dedicated analysis software developed by the authors^11,21,22^.

### Tracking watermelon growth after obtaining the head shape

From the ten produced head shapes, four watermelons were selected for tracking their growth for several days after removing them from the mold to assess whether their growth ability was hindered in some form. Daily scans were made, and three growth-related measurements were performed: circumference, biparietal diameter, and fronto-occipital diameter. These measurements were chosen because they are commonly employed in clinical settings to diagnose head deformities.

#### Ethics statement

All methods were carried out in accordance with relevant guidelines and regulations. The data were collected as part of standard clinical care during hospital treatment. The MRI scanner used is an approved medical device used for routine clinical care. The study received ethical approval, and the informed consent was waived by the ethics committee of the Medical Faculty Carl Gustav Carus at the Technical University of Dresden to analyze the routine clinical data (study number EK 261082012).

## Results

### Comparing the reshaped watermelons with the original head scans

**Fig. 7:**
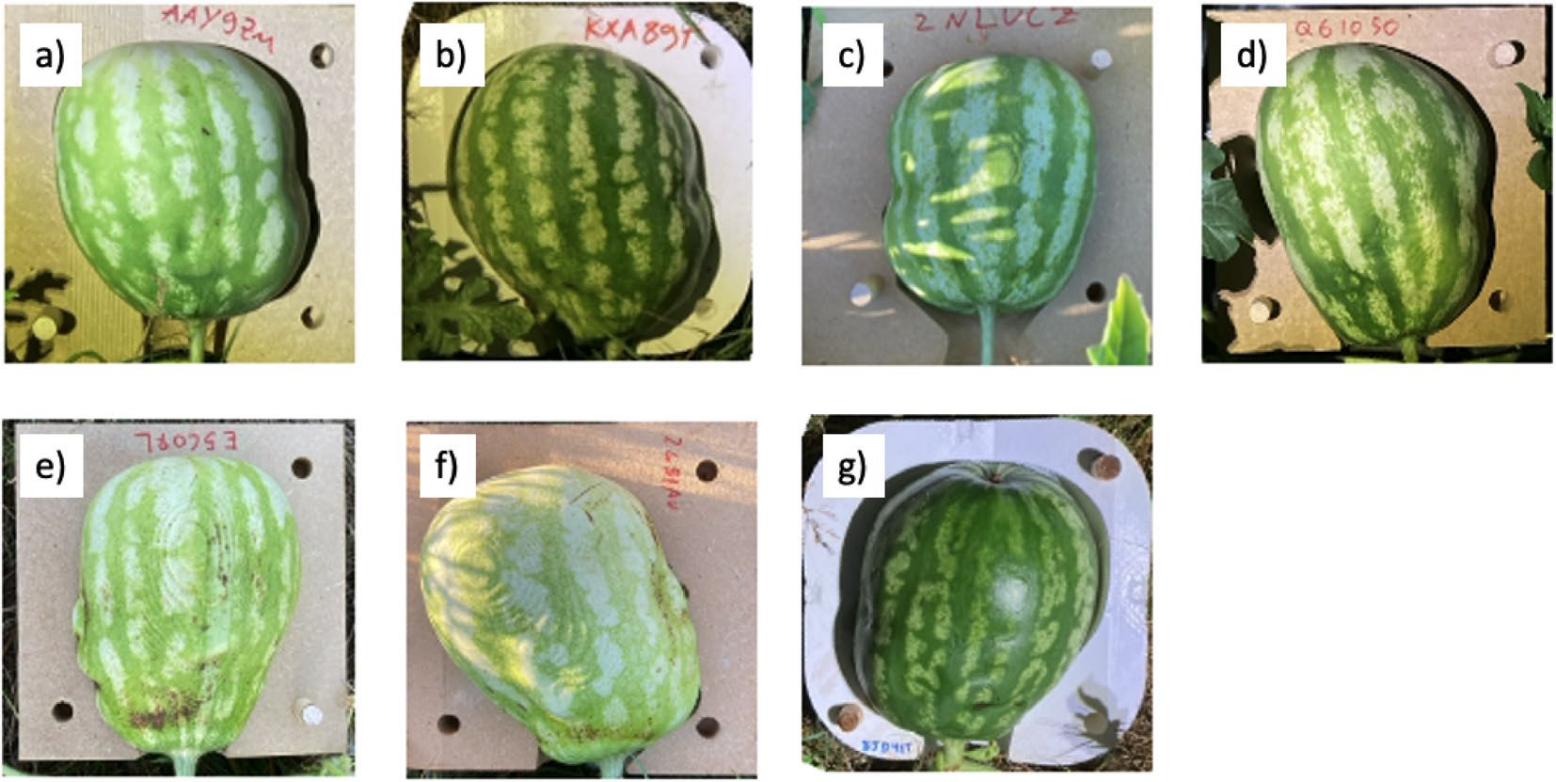
Resulting head shapes molded on the watermelons, shown inside the molds.

In Fig. 7, some of the resulting head shapes molded on the watermelons are presented. After the volume of the mold was filled, it was removed, and the shapes were scanned as described in Section 2 F. Four of the watermelons when removed from the molds, were allowed to grow freely after the initial scan, and their shapes were scanned daily for several days. This was meant to study the effect on the subsequent growth of the watermelons after being shaped by the molds.

We observed no differences in watermelon growth between the two mold materials. Both MDF and PLA offered sufficient rigidity to support the growth process and were readily available for fabrication at our research facilities. In both cases, the watermelons filled the mold cavities, resulting in similarly precise shapes.

**Fig. 8:**
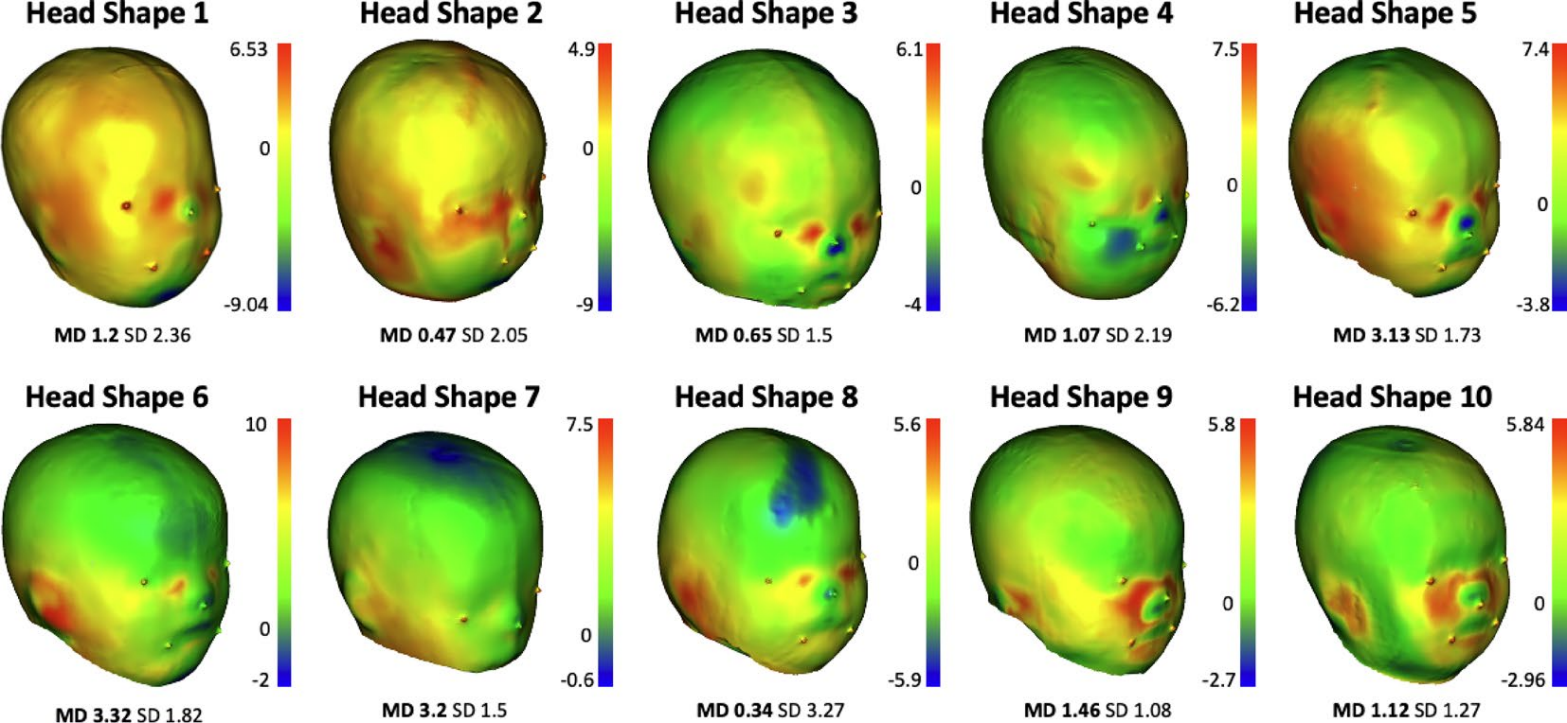
Measurement of the distances between the original head scans and the watermelon shape created with the mold. Medium distance (MD) and standard deviation (SD) are in millimeters. Images generated with Cloud Compare *software* (version 2.12.4) [GPL software] (2021) retrieved from http://www.cloudcompare.org/).

In Fig. 8, the distance measurements between the original 3D head scans and the corresponding produced watermelon shapes are presented. The mean distance between all watermelon head shapes and their original infant head scans was 1.6 mm (SD 1.88 mm).

The individual values of the CR and CVAI are presented in Table I. The average CR values of the ten original 3D scans and ten watermelon head shapes are 88.97% (SD 5.24%) and 91.11% (SD 5.04%), respectively, and the average CVAI values are 5.54% (SD 3.3%) in the original scans and 4.7% (SD 3.07%) in the watermelons.

### Measurement of growth after the molding of the watermelons

**Fig. 9:**
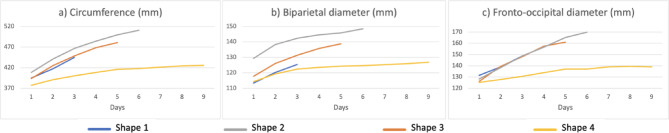
Daily measurements of four watermelons during growth after molding. **a**) the circumference, **b**) biparietal diameter, and **c**) fronto-occipital diameter.

The measurements of the circumference, biparietal diameter, and fronto-occipital diameter (in mm) of the watermelons that continued to grow after reaching the head shape are presented in Fig. 9.

## Discussion

In this study, a novel partial biological phantom model of infant head shapes is presented. After daily measurements, we observed that this biological model was able to continue growing in all directions after the initial shaping process, as depicted in Fig. 9. The natural growth of watermelon is a key feature, as it mimics one of the aspects of the dynamic nature of infants’ cranial development. Additionally, watermelons maintain their capacity to be reshaped, creating an experimental platform for the development of dynamic cranial remolding orthoses.

**Table 1:** Distances between the original scans and the produced watermelon shapes (mm) and standard deviation (SD) (mm).

	CR (%)		CVAI (%)	
Shape ID	Original	Watermelon	Original	Watermelon
1	92.9	90.1	7.8	6.9
2	94.2	98.2	2.4	2.4
3	91.7	91.7	9.8	9.9
4	84	86	7.5	6
5	89.7	93.8	5.5	4.6
6	84.2	89.3	5.9	5.2
7	82.3	87.6	6.5	4.3
8	82.9	84.3	8.8	7.2
9	97	100.1	0.6	0.5
10	90.8	90	0.6	0.04
**Average**	**88.97**	**91.11**	**5.54**	**4.7**

According to our experiments, the production of watermelons with specific head shapes is an accessible and straightforward process, with a 1.6 mm average distance achieved in approximately six weeks. This is below the average measurement error observed by Aarnivala et al., who found variations of up to 3 mm in the measurements of the diagonal differences of infant heads in the clinical context^20^. The high growth speed of the watermelons makes the experiment feasible and shaping a watermelon into a specific head shape can take only a few days. The symmetry-related measurement differences between the original scans and the watermelons are 2.14% in the CR and 0.84% in the CVAI. The similarity of these measurements was important to obtain since the presented model is intended to be used in experiments with novel dynamic cranial remolding orthoses for DP, where watermelons with asymmetrical head shapes are remolded into symmetrical shapes while growing inside the orthoses.

Current skull growth models include computational simulations, statistical models, or additively manufactured physical models with inflatable balloons to mimic head growth. The introduction of a biological model with growth and deformability characteristics could contribute to improved research, where the dynamic, mechanical interactions of a cranial orthosis with a growing skull need to be studied.

The watermelons were immediately removed from the molds once fully shaped and were never returned to the molds afterward, as they continued growing. The sole purpose of the molds was to give the watermelons the shape of the original asymmetric infant heads MRI scans while maintaining their health and allowing them to continue growing. This will enable in future work their subsequent placement inside customized lattice structures, which will reshape them into more symmetric forms during growth, thus bringing two key aspects of the mechanical behavior of the human head for interaction with the lattice structures: growth and the ability to be reshaped by a constraining helmet.

### Limitations

We acknowledge some limitations derived from the nature of this phantom. The absence of bone plates and sutures in this model means that there is no possibility of reproducing the characteristic growth dynamics of these structures during growth in infants, making this one of the main limitations of the presented model.

Some anatomical features, such as the nose, the region surrounding the mouth, and the ear lobes, were not able to reproduce accurately, as shown in Fig. 8. This was due to the natural limitation of reproducing the minimum curvatures by the watermelon surface when growing inside the mold. Moreover, during the design phase of the molds, these specific areas in the molds were smoothed for better surface finishing results. The remaining regions experienced no visible alterations.

Due to time and logistical constraints, we were unable to duplicate the ten head shapes with additional watermelons. This was primarily due to the limited number of available molds, and to the ripening season, the limited time window of ideal temperature conditions necessary for successful cultivation.

The purpose of employing a biological growth model that mimics the human head is not to investigate or draw conclusions about human growth itself, but rather to provide a development and testing platform for the functionally graded lattice structures in Fig. 2d), intended to guide head growth in a dynamic and controlled manner.

## Conclusion and future works

Current cranial remodeling orthoses rely on the mechanical principle of growth restriction; however, the use of rigid helmets often leads to complications, particularly due to concentrated pressure points as the infant’s head grows. Through this study, our objective is to establish an experimental platform that can help explore different lattice configurations, functioning as the inner lining of the helmets to help mitigate such complications.

This innovative approach, which employs molded watermelons and digital fabrication techniques, represents a preliminary step in the study of the mechanical interactions of cranial orthoses with growing heads during the treatment of cranial deformities in infants. This study provides a conceptual introduction for further exploration and refinement, with the inclusion of bone plates and sutures being a logical and promising next step in our pursuit of a more accurate and clinically relevant biological model. Ultimately, this research has the potential to positively impact the field of the development of novel cranial orthoses using functionally graded lattice structures.

## Data Availability

The authors confirm that the data supporting the findings of this study are available within the article and/or upon request, by sending an email to the corresponding author, fveloso@ipca.pt.
